# Neurological Presentations of Endemic Tropical Diseases in Central Europe

**DOI:** 10.3390/jcm14207393

**Published:** 2025-10-20

**Authors:** Anna Gójska-Grymajło, Beata Biernat, Katarzyna Sikorska

**Affiliations:** 1 Department of Planned Neurology, Institute of Maritime and Tropical Medicine, University Center of Maritime and Tropical Medicine, Medical University of Gdańsk, 81-519 Gdynia, Poland; annagojska@gumed.edu.pl; 2 Division of Tropical Parasitology, Institute of Maritime and Tropical Medicine, Medical University of Gdańsk, 81-519 Gdynia, Poland; beata.biernat@gumed.edu.pl; 3 Division of Tropical and Parasitic Diseases, Institute of Maritime and Tropical Medicine, Medical University of Gdansk, 81-519 Gdynia, Poland

**Keywords:** tropical diseases, neurological complications, mosquitoes spread, West Nile fever, dengue, chikungunya

## Abstract

Climate change increases the risk of tropical diseases—previously rare in Central Europe—emerging as local or zoonotic infections, not just imported cases. Diagnosing such illnesses without a travel history is challenging, especially given their atypical presentations and potential for neurological complications. We highlight the recent spread of mosquito vectors and focus on West Nile fever, dengue, and chikungunya, discussing their typical symptoms and possible nervous system involvement, emphasizing the need for heightened awareness among neurologists in our region.

## 1. Introduction

**Figure 1:**
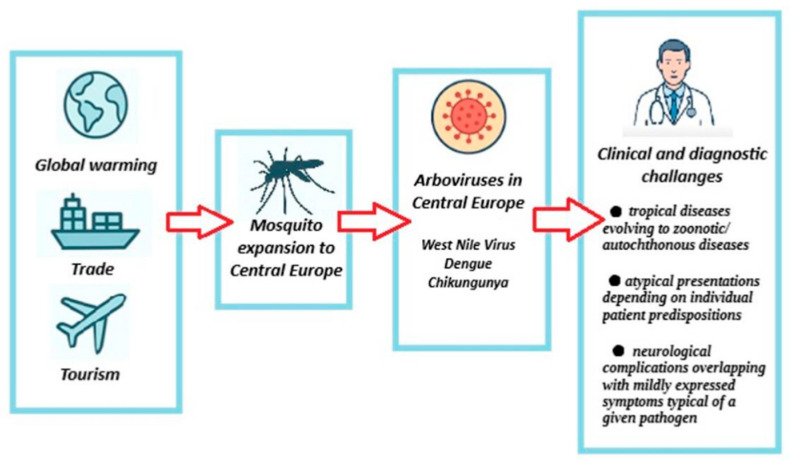
Processes contributing to the emergence of clinical and diagnostic challenges related to tropical diseases in Central Europe. In our opinion, West Nile virus, dengue and chikungunya are most likely to appear in this region as autochthonous diseases.

Climate change has always influenced the epidemiology of infectious diseases, including tropical diseases. Current climate shifts cause the tropical diseases—previously unseen in Central Europe, including Poland—to have the potential to emerge not only as cases imported by travelers but also as zoonotic and autochthonous infections. The greatest risk is associated with vectors such as mosquitoes and birds. Diagnosing unusual illness in a patient without a history of recent travel to tropical regions can be challenging. Furthermore, many tropical diseases may present atypically depending on individual predispositions. In some cases, patients may develop serious neurological complications that overlap with mildly expressed symptoms typical of a given pathogen (Figure 1).

In light of this, we present interesting data on the expansion of mosquito populations in recent years—a key reason to consider tropical diseases in neurological diagnostics in Central Europe. Next, we discuss tropical diseases such as West Nile fever, dengue fever, and chikungunya virus infection, which, in our opinion, are most likely to appear in our geographic zone—along with their most common symptoms and neurological complications.

## 2. Mosquitoes

Among all hematophagous insects, mosquitoes are the most epidemiologically and medically significant arthropods due to their aggressiveness, widespread presence, and ability to transmit various pathogens. Diseases spread by mosquitoes are becoming increasingly common due to the global impact of climate change, which significantly affects both health and the economy worldwide. The primary risk for the spread of invasive vector species is human movement (e.g. increased tourism) and trade. Most invasive species were introduced to Europe via international trade in used tires, where female mosquitoes lay their eggs. Moreover, the use of cargo containers for intercontinental shipping has further increased the chance of new species being introduced. Additionally, anthropogenic changes—such as global warming, urbanization, and environmental pollution—significantly contribute to their spread [1]. Urban areas, in particular, due to their landscape structure, lack of natural regulatory mechanisms, and loss of biodiversity, are especially vulnerable to colonization by invasive species [2]. Knowing the current mosquito distribution in a given area is essential to assess the potential health risks to humans and animals—especially for invasive species due to their ability to transmit tropical pathogens [3].

The native and most common mosquito species in Europe, including Poland, is *Culex (Culex) pipiens complex*. It is found in all European countries except Iceland and the Faroe Islands and is widely distributed in the temperate zones globally. This taxon consists of two forms (biotypes): *Cx. pipiens pipiens* (mainly ornithophilic and exophagic) and *Cx. pipiens molestus* (mainly anthropophilic and endophagic). Female *Cx. pipiens* feed on the blood of various vertebrates, allowing them to contribute to the West Nile virus (WNV) amplification cycle among birds and transmit viruses to humans and other mammals. Both forms demonstrate high ecological flexibility and multiple generations per year. Mated females overwinter, which provides a significant opportunity for the pathogen to survive in the vector during winter [4,5].

Invasive mosquito species in Europe include those from the genus *Aedes (Stegomyia)*. These insects are believed to transmit over 300 arboviruses, around 100 of which affect humans [6], including dengue and chikungunya viruses discussed in this article.

*Aedes (Stegomyia) albopictus*, originating from Southeast Asia’s tropical forests, has spread globally. This geographic expansion occurred primarily over the past three decades through passive transport of eggs in used tires or bamboo—with bamboo being the route of import to Belgium and the Netherlands. Passive transport via ground vehicles is believed to be the main pathway for introducing *Ae. albopictus* to southern France, Germany, the Balkans, the Czech Republic, Spain, and Switzerland. The first report of this species being imported into Europe came from Albania in 1979. Although *Ae. albopictus* became established in Albania, no reports emerged from other European countries until 1990, when it was discovered in Italy. Italy is now the most affected country in Europe, with the highest prevalence in the Veneto and Friuli-Venezia-Giulia regions, large areas of Lombardy and Emilia-Romagna, and coastal areas of central Italy. In 1999, the species was recorded in France, and in 2000 in Belgium [7].

This mosquito displays aggressive and troublesome behavior during the day, when females search for hosts.

*Aedes (Stegomyia) aegypti*

This African mosquito historically spread across continents via ships and was established in southern Europe from the late 18th to the mid-20th century. Its disappearance from the Mediterranean, Black Sea, Canary Islands, Madeira, and Azores regions remains unexplained. Since then, it has recolonized Madeira and reappeared in parts of southern Russia and Georgia (Krasnodar and Abkhazia). It has reportedly been introduced to the Netherlands, Canary Islands, and Cyprus. The species is now widespread across large parts of Georgia, including the capital, Tbilisi [8].

Today, it is one of the most widespread mosquito species in the world.

*Aedes (Ochlerotatus) japonicus japonicus*

*Ae. japonicus* is the third invasive mosquito species recorded in Europe. Native to Korea, Japan, Taiwan, southern China, and Russia, it has since spread to many other countries, starting in the 1990s. This geographic spread was also facilitated by the above-mentioned international trade in used tires. Its range in Central Europe continues to expand. *Ae. japonicus* was first reported in Europe in 2000 when it was detected in Normandy (Orne), northern France. It was then reported in Belgium in 2002 at a tire storage site, with adult and larval presence confirmed in 2007 and 2008. It was detected in Switzerland in 2008, and later studies found a colonized area of 1400 km, including regions of Germany—marking the first detection of an invasive mosquito spreading in Central Europe. Adult *Ae. japonicus* were subsequently found in southern Germany in 2011 [9].

*Aedes (Ochlerotatus) koreicus*

*Ae. koreicus* was discovered in 2008 in a small area of eastern Belgium, though it does not appear to be spreading. The species was also recently detected in the Veneto province of Italy, but further monitoring is needed to evaluate its spread. Entomological studies from 2011 concerning its colonization in Veneto did not reveal the routes of introduction [10].

## 3. Viruses

Viruses that maintain transmission cycles between vertebrate animal reservoirs as primary amplifying hosts and arthropods as primary vectors are known as arboviruses (arthropod-borne viruses). Arboviruses must replicate within their vectors before transmission. Female mosquitoes acquire the virus while feeding on the blood of an infected animal; the virus then replicates in the mesenchymal–epithelial cells, subsequently infecting the salivary glands after secondary amplification in other cells and tissues. The virus is then released from the epithelial cells of the salivary glands and transmitted during blood-feeding on a vertebrate host [11].

Below, we present three tropical diseases caused by arbovirus infection, which, due to the aforementioned environmental changes, are most likely to become endemic in Central Europe.

### 3.1. West Nile Fever

#### 3.1.1. Epidemiology

West Nile virus (WNV) is one of the most widespread arboviruses in the Old World, throughout Africa, the Middle East, parts of Europe, and the former Soviet Union, South Asia, and Australia. The virus was first isolated from a human in Uganda in 1937. In Europe, circulation of the virus in its natural reservoirs likely began in the 1950s. WNV has been and continues to be introduced into Europe by migratory birds from sub-Saharan Africa, North Africa, or the Middle East. Active WNV transmission occurs during the mosquito activity season—from spring to autumn—with most human and equine infections recorded between July and September. The first epidemic outbreak in Europe occurred in southern France (Camargue) in 1962–63, and the first major epidemic outbreak (400 cases) was recorded in Romania in 1996 [12].

From 2010 to 2018, between 110 and 991 neuroinvasive disease cases were reported in EU countries [13]. Among EU countries, Greece, Italy, and Romania reported the most cases. In the years 2012–2021, a total of 3632 cases of WNV were reported from European countries. For Central Europe Austria, Croatia, Czechia, Germany, Hungary, and Romania reported autochthonous human cases of WNV infection with an annual incidence ≥0.1/100,000. Bulgaria, Slovakia and Slovenia reported few autochthonous human infections with WNV and annual rates were lower than 0.1/100,000 population [14].

There is no specific treatment for WNV infection; therapy is symptomatic only. A vaccine is also not yet available.

#### 3.1.2. Mode of Transmission

Birds are the natural reservoir of the virus. Virus circulation primarily occurs between birds and mosquitoes of the *Culex* genus. The virus can overwinter in mosquitoes. Mammals, including humans, are incidental and terminal hosts. Human infection most often results from the bite of an infected mosquito and only rarely from organ transplantation, blood transfusion, or breastfeeding [15].

#### 3.1.3. Typical Course of Illness

Typical general symptoms of the disease and possible neurological symptoms are presented in Table 1.

The majority (approx. 80%) of infected individuals will not develop any symptoms. The incubation period typically ranges from 3 to 14 days (up to 21 days in cases of immune deficiency).

Typical symptoms are unfortunately indistinguishable from dengue or other viral infections. These include nonspecific flu-like symptoms such as fever, headache, muscle pain, nausea, vomiting, and loss of appetite [15]. The symptoms are usually self-limiting and last 3–10 days. Despite the name of the disease, many patients do not exhibit elevated body temperature [16]. Generalized lymphadenopathy occurs in approximately 20–25% of patients, though this rate has been clearly lower in recent outbreaks. Eye pain, sore throat, abdominal pain and diarrhea may also occur.

A rash occurs in about 25–50% of patients, most commonly on the chest, back and arms. It is morbilliform or maculopapular, sometimes itchy, and may be accompanied by dysesthesia. Interestingly, the presence of a rash has been associated with a lower risk of neurological complications and death [17,18].

#### 3.1.4. Neurological Symptoms and Complications

Only 0.4–0.6% of patients develop neurological symptoms. However, the mortality risk for those with neurological involvement is 10%. Factors that predispose to neurological presentation include older age, cancer (especially hematological), organ transplants, and genetic predisposition (e.g., )deficiency) [19,20]. Factors increasing mortality risk include older age, male sex, altered mental status, diabetes, coronary artery disease, hepatitis B infection, alcohol abuse, and immunosuppression [21,22].

The most common neurological manifestations of infection are meningitis (more frequent in children), encephalitis (more frequent in older adults), and acute flaccid paralysis.

**Meningitis** symptoms are identical to other viral meningitis cases: sudden onset of fever and headache, photophobia and phonophobia, and meningeal signs on neurological exam. CSF analysis reveals moderate lymphocytic pleocytosis (usually fewer than 500 cells/mm^3^). However, early sampling may show neutrophil predominance. Prognosis is usually good, though some patients experience chronic headaches, fatigue and muscle pain [23].

**Encephalitis** can range in severity from mild delirium to severe encephalopathy, leading to coma and death. WNV-related encephalitis may present with various neurological syndromes, with extrapyramidal symptoms (hypomimia, bradykinesia, and postural instability) being suggestive of WNV infection. Patients often develop bilateral low-frequency tremor of the upper limbs. Myoclonuses of the upper limbs and face are common and they often persist during sleep. Cerebellar symptoms (ataxia, trunk asynergia and gait disturbances) have also been observed. These motor symptoms usually follow consciousness disturbance and usually resolve during recovery, though Parkinsonian features and tremor may persist in severe cases [23,24].

On MRI, encephalitis or meningoencephalitis secondary to WNV infection may show nonspecific findings. Lesions may appear later in the course of the disease or not at all, warranting repeated imaging. Lesions most commonly appear in the basal ganglia and thalami but may also affect the white and gray matter and brainstem. The only finding suggestive of WNV is the symmetric involvement of the basal ganglia, which is typical of flaviviruses [25,26].

**Acute flaccid paralysis** results from viral attack on spinal motor neurons, mimicking poliomyelitis. Symptoms typically appear within 24–48 h from disease onset and begin suddenly—usually as asymmetric limb weakness (including monoplegia), necessitating stroke differentiation. Quadriplegia and facial muscle involvement may occur. Sensory symptoms are typically absent though patients may experience intense limb pain before or at the onset of paralysis. In some cases, this pain may persist [23,27].

WNV infection may also involve ophthalmic symptoms which are among the most frequent after fever and neurological manifestations. Most common are chorioretinitis, retinal hemorrhages, and vitreitis [28].

#### 3.1.5. Pathogenesis of Neurological Symptoms

The virus primarily replicates in dendritic skin cells. About a week after inoculation, WNV migrates to the central nervous system (CNS) and crosses the blood–brain barrier through mechanisms not yet fully understood. Higher viremia increases the risk of CNS invasion. Elevated levels of proinflammatory cytokines (interleukin-6 (IL-6) and tumour necrosis factor- alpha (TNF-alpha)) also contribute to blood–brain barrier disruption. Neurons, astrocytes, and microglial cells are affected—neurons die rapidly, also likely due to microglial activation, while astrocytes are infected more slowly and serve as sites for continued viral replication [29,30].

### 3.2. Dengue

#### 3.2.1. Epidemiology

Dengue is the most commonly diagnosed arboviral disease. It occurs endemically and periodically in epidemic outbreaks within the subtropical and tropical zones, primarily in urban and peri-urban areas, and its range is expanding rapidly. Severe dengue was first recognized in the 1950s during outbreaks in the Philippines and Thailand. Before 1970, only nine countries had reported epidemic outbreaks of severe dengue. Currently, nearly half of the global population lives in areas at risk of infection. Dengue was listed among the top 10 global public health threats by the WHO in 2019.

A concerning trend is the steadily increasing number of autochthonous (locally acquired) dengue cases in Mediterranean European countries, mainly France, Spain, and Italy. The first confirmed autochthonous case in Europe occurred in 2010 in Croatia. In 2022, local transmission unrelated to travel accounted for 71 cases, rising to 304 cases which were reported from France, Italy and Spain in 2024 [31].

There are no specific antiviral treatments for dengue, and management is purely symptomatic. The case fatality rate does not exceed 1%, but it depends on access to medical care. Currently, two vaccines are available in Europe: CYD-TDV (Dengvaxia by, Sanofi Pasteur, France), which is only recommended for individuals with prior DENV infection, and TAK-003 (Qdenga by Takeda, Japan). According to the WHO statement, TAK-003 is recommended in children aged 6–16 years in settings with high dengue transmission intensity. Persons living in non-endemic countries who have previously been infected with any of the four dengue virus serotypes following travel to dengue-endemic countries may benefit from TAK-003 vaccination to prevent a second (and hence potentially more severe) dengue infection when traveling again to an endemic country [32].

#### 3.2.2. Mode of Transmission

As with West Nile virus, mosquitoes are the vector, but in the case of dengue, it is the Aedes group. Furthermore, humans serve as the natural reservoir for disease transmission, except in Southeast Asia and West Africa, where mosquitoes can transmit the virus to non-human primates, bats, and small rodents. Virus amplification occurs in humans during the viremic phase, when infected individuals are bitten by mosquitoes. Interestingly, infected mosquitoes remain infectious for their entire lifespan (30–45 days) and can pass the infection to the next generation by laying infected eggs [33].

There are four types of dengue virus (DENV-1, DENV-2, DENV-3, and DENV-4). The risk of developing severe dengue is highest in individuals experiencing a second infection with a different DENV type than in the first infection (secondary or heterotypic infection) [34].

#### 3.2.3. Typical Disease Course

The typical course of illness and possible neurological complications are shown in Table 2.

According to the 2009 WHO classification, the following categories/stages of dengue infection are identified:

**-**  **Dengue without warning signs**—fever and two of the following: nausea/vomiting, rash, headache, pain in the eyes, muscles or joints, leukopenia, positive tourniquet test (petechiae after pressure from a blood pressure cuff)
**-**  **Dengue with warning signs**—symptoms as above plus one or more of the following: abdominal pain or tenderness, persistent vomiting, fluid accumulation (ascites, pleural effusion), mucosal bleeding, lethargy or restlessness, hepatomegaly > 2 cm, increase in hematocrit concurrent with a rapid drop in platelet count
**-**  **Severe dengue infection**—at least one of the following: severe plasma leakage (leading to shock or pleural effusion with respiratory failure), severe bleeding (as assessed by a clinician), severe organ damage (AST or ALT > 1000 U/L, impaired consciousness, organ failure)

Uncomplicated illness typically lasts several days to two weeks. Recovery from severe disease can take several months.

#### 3.2.4. Neurological Symptoms and Complications

The nervous system may be involved in any stage of the disease. Neurological symptoms may occur even in the absence of typical DENV infection symptoms—up to 50% of patients with encephalitis show no typical signs.

The most common neurological complications include encephalopathy (headaches, altered levels of consciousness) and encephalitis (0.5–6.2% of patients), which presents with altered mental status, headaches, seizures, fever, nausea, vomiting, and focal neurological deficits [35].

Other neurological syndromes potentially associated with DENV include meningitis, stroke, myositis, hypokalemic paralysis, mononeuropathies (optic neuritis, Bell’s palsy, long thoracic nerve neuropathy, phrenic nerve neuropathy), polyneuropathies, Guillain–Barré syndrome (during or after infection), transverse myelitis (very rare; occurs during or after infection), ADEM (in recovery phase, after the febrile stage; effectively treated with intravenous methylprednisolone pulses), and Parkinsonism (21 documented cases) [35,36].

While most of these neurological syndromes are well known to neurologists and their clinical presentation does not significantly differ from the same syndromes caused by other etiologies, hypokalemic paralysis is noteworthy. It presents with sudden flaccid paralysis of all four limbs, without facial muscle or sphincter involvement. Diagnosis is confirmed by hypokalemia of 3 mmol/L or less. The paralysis typically occurs between the 2nd and 5th day of fever and develops over 4–24 h. The pathogenesis is unclear but symptoms resolve rapidly and completely after supplementation with small doses of potassium [35].

#### 3.2.5. Pathogenesis of Neurological Symptoms and Complications

Initially, DENV was considered non-neurotropic, but autopsy studies of fatal cases have confirmed its invasiveness. Encephalitis, meningitis, and myelitis are most commonly caused by DENV-2 and DENV-3, although DENV-1 and DENV-4 have also been found in encephalitis cases [35,37]. Mechanisms of damage vary depending on the complication. Encephalitis results from direct viral invasion of the nervous system. Autoimmune responses are responsible for Guillain–Barré syndrome, ADEM, transverse myelitis, mononeuropathies and polyneuropathies. Metabolic disturbances and systemic infection underlie encephalopathy, stroke, hypokalemic paralysis, myositis, and rhabdomyolysis [35,38].

#### 3.2.6. Neuroradiological Findings

In encephalopathy, MRI/CT usually shows no abnormalities or only diffuse cerebral edema. In encephalitis, CT may show microhemorrhages and hypodensity in the thalamus and basal ganglia. MRI (T2, DWI/ADC, SWI sequences) may reveal hyperintense changes in the basal ganglia, thalamus, temporal lobes, hippocampus, and less commonly in the brainstem and cerebellum, as well as symmetric gyri swelling. Unfortunately, similar imaging patterns are also observed in ADEM, Japanese encephalitis, and Chikungunya virus infection [35,36,39].

Interestingly, while radiological findings may resemble Japanese encephalitis (in which one-third of patients die and around 50% suffer chronic neurological sequelae), patients with DENV-associated encephalitis, despite pronounced neurological symptoms, generally recover with little or no residual deficits [35].

### 3.3. Chikungunya

#### 3.3.1. Epidemiology

The Chikungunya virus (CHIKV) was first identified in humans in 1952. Since 2004, rapid expansion to over 60 countries across all continents has been observed. Since 2007, after first reports from Italy and France, local transmission of CHIKV infections has been monitored in European countries. In 2024, approximately 480,000 cases of CHIKV and over 200 deaths were reported worldwide. The majority of cases have been reported in South and Central America (Brazil, Paraguay, Argentina, and Bolivia) [40].

From 2018 to 2022, European countries reported 693 cases of CHIKV, among them 37 imported cases from Central Europe (Czechia, Hungary and Poland) and 631 autochthonous cases from France and Italy. In Europe (excluding the outermost regions), in years 2007–2025, cases of autochthonous cases of CHIKVD have been reported only from France and Italy. The number of autochthonous cases ranged from 2 in 2010 to 1253 in 2025 [41,42].

There is no specific treatment for CHIKV infection and only symptomatic management is possible. In the US and Europe, one type of live-attenuated vaccine (VLA1553, or IXCHIQ by Valneva SE, France) is available.

#### 3.3.2. Mode of Transmission

The virus is mainly transmitted through the bites of mosquitoes from the Aedes group (*A. aegypti* or *A. albopictus*). Humans and animals (mainly primates) serve as reservoirs for the virus. Larger outbreaks outside of Africa are sustained by mosquitoes transmitting the virus between infected individuals [43]. Very rare cases of transmission through blood products have been reported [44]. Transplacental transmission can lead to miscarriage, and the highest risk of fetal infection is associated with symptomatic maternal infection during the perinatal period [45].

#### 3.3.3. Typical Course of Illness

The typical course of the disease and potential neurological complications are presented in Table 3.

Unlike many viruses, CHIKV infection is usually symptomatic (about 70% of infected individuals). The incubation period ranges from 1 to 12 days. The primary symptom is symmetrical arthritis with severe joint pain (which gave the disease its name, referring to a “stooped walk”) and high fever. Other common accompanying symptoms include rash, gastrointestinal issues, weakness, and headaches. Symptoms typically last 1–2 weeks [46]. Chronic rheumatologic complications affect up to half of patients [47].

#### 3.3.4. Neurological Complications

Neurological complications in CHIKV infection are rare (9–16% of symptomatic patients), but an increase has been observed over the past decade. These include, in order from most to least common: encephalopathy, meningoencephalitis, myelitis, Guillain–Barré syndrome, and less frequently, seizures (mainly in children) and cranial nerve neuropathies (involving cranial nerves II and III). Neurological symptoms do not differ clinically in these symptoms of other causes. However, one helpful diagnostic clue is that neurological complications usually coincide with the acute phase of infection (on average 10 days after symptom onset), while Guillain–Barré syndrome typically appears shortly after the acute phase [48,49,50,51]. Although neurological complications are rare, they represent a major cause of CHIKV mortality. They account for up to 25% of atypical cases, as much as 60% of severe atypical cases, and are the primary reason for admission to intensive care units [52].

#### 3.3.5. Pathogenesis of Neurological Complications

The mechanisms by which CHIKV interacts with the CNS and contributes to neurological disorders remain poorly understood. Nervous system damage due to CHIKV infection is a mix of direct viral infection, possibly through the choroid plexus, and excessive activation of the host immune system, triggering neuroinflammation [48,53]. Asian and ECSA (East/Central/South African) strains can spread in astrocytes and neurons, with the Asian strains appearing more virulent, causing greater mortality through upregulation of pro-apoptotic gene expression [47]. Interestingly, recent reports suggest that the neuroinflammatory processes, with proinflammatory cytokine release and activation of receptors such as high mobility group box 1 (HMGB1), tumor protein P63, and signal transducer and activatior of transcription 3 (STAT3), are similar to those seen in various neurological conditions, including autism, cerebral palsy, depression, Alzheimer’s disease, and neuromyelitis optica [53].

#### 3.3.6. Neuroradiological Findings

MRI findings in cases of encephalitis are often nonspecific, and in many cases no changes are observed. However, in cases of myelitis, one study found a significant portion of patients presenting a “clock face” appearance in transverse spinal cord sections. Other findings included thickening and enhancement of the anterior spinal roots and numerous hyperintense lesions in the white matter of the spinal cord, particularly in the medulla oblongata [50,54].

## 4. Conclusions

Climate change and global trade are contributing to the spread of invasive mosquito species capable of transmitting tropical diseases in Central Europe. This increases the risk of locally acquired infections and highlights the need to consider tropical pathogens in neurological diagnostics.

## Figures and Tables

**Table 1:** West Nile virus infection—typical general symptoms and most likely neurological manifestations.

Typical Systemic Symptoms	Neurological Symptoms/Complications (Most Common)
– incubation: 3–14 days	– Meningitis (symptoms typical for regular viral meningitis)
– typical symptoms (3–10 days):	– Encephalitis (low-frequency upper limb tremor, myoclonus of upper limbs and face, Parkinsonian features, cerebellar symptoms)
flu-like (fever, headache, muscle pain, nausea, vomiting, loss of appetite)	– Acute flaccid paralysis (mimics poliomyelitis; sudden asymmetric limb paresis, sometimes mimicking stroke)
rash (25–50% of patients)	
generalized lymphadenopathy (rare)	
eye pain, sore throat, abdominal pain, diarrhea (rare)	

**Table 2:** Typical course of the disease and possible neurological complications in the course of Dengue virus infection.

Typical Course of the Disease	Possible Neurological Complications
Dengue without warning symptoms—fever and two of the following: nausea/vomiting, rash, headache, eye, muscle or joint pain, leukopenia, positive band test Dengue with warning symptoms—symptoms as in point 1 plus any of the following symptoms: abdominal pain or tenderness, persistent vomiting, ascites, pleural effusion), bleeding from mucous membranes, lethargy or agitation, hepatomegaly > 2 cm, increased hematocrit with a simultaneous rapid decrease in platelet count. Severe dengue virus infection—at least one of the following symptoms: severe plasma loss (secondary to shock or effusion with concomitant respiratory failure), severe bleeding, severe organ damage	Most often: − **encephalopathy:** headaches, quantitative disturbances of consciousness; – **encephalitis** (0.5–6.2% of patients): quantitative and qualitative disturbances of consciousness, headaches, seizures, fever, nausea, vomiting, focal neurological deficits. Less common: – meningitis; – stroke; – myositis; – hypokalemic paralysis; – mononeuropathies (inflammation of nerve II, Bell’s palsy, neuropathy of the long thoracic nerve, neuropathy of the phrenic nerve); – polyneuropathy; – Guillain–Barré syndrome; – ADEM; – transverse myelitis (very rare); – Parkinsonism (very rare).

**Table 3:** Typical course of illness and possible neurological complications in Chikungunya virus infection.

Typical Course of Illness	Possible Neurological Complications
- Incubation period: 1–12 days - Typical symptoms (1–2 weeks): symmetrical arthritis with severe joint pain and high fever	Most common: - Encephalopathy - Meningoencephalitis - Myelitis - Guillain–Barré syndrome Rare: - Seizures (mainly in children) - Cranial nerve neuropathies

## Data Availability

Not applicable.

## References

[1] Giunti G., Becker N., Benelli G. (2023). Invasive mosquito vectors in Europe: From bioecology to surveillance and management. Acta Trop..

[2] Gierek M., Ochała-Gierek G., Woźnica A.J., Zaleśny G., Jarosz A., Niemiec P. (2024). Winged Threat on the Offensive: A Literature Review Due to the First Identification of *Aedes japonicus* in Poland. Viruses.

[3] Jawień P., Pfitzner W.P., Schaffner F., Kiewra D. (2024). Mosquitoes (Diptera: Culicidae) of Poland: An Update of Species Diversity and Current Challenges. Insects.

[4] Muñoz J., Ruiz S., Soriguer R., Alcaide M., Viana D.S., Roiz D., Vázquez A., Figuerola J. (2012). Feeding patterns of potential West Nile virus vectors in South-West Spain. PLoS ONE.

[5] Rizzoli A., Bolzoni L., Chadwick E.A., Capelli G., Montarsi F., Grisenti M., de la Puente J.M., Muñoz J., Figuerola J., Soriguer R. (2015). Understanding West Nile virus ecology in Europe: *Culex pipiens* host feeding preference in a hotspot of virus emergence. Parasites Vectors.

[6] Biernat B. (2023). Choroby zakaźne: Wektory przenoszące drobnoustroje: Komarowate. Interna Szczeklika.

[7] European Centre for Disease Prevention and Control, An Agency of the European Union Aedes albopictus—Factsheet for Experts. https://www.ecdc.europa.eu/en/disease-vectors/facts/mosquito-factsheets/aedes-albopictus.

[8] European Centre for Disease Prevention and Control, An Agency of the European Union Aedes aegypti—Factsheet for Experts. https://www.ecdc.europa.eu/en/disease-vectors/facts/mosquito-factsheets/aedes-aegypti.

[9] European Centre for Disease Prevention and Control, An Agency of the European Union Aedes japonicus—Factsheet for Experts. https://www.ecdc.europa.eu/en/disease-vectors/facts/mosquito-factsheets/aedes-japonicus.

[10] European Centre for Disease Prevention and Control, An Agency of the European Union Aedes koreicus—Factsheet for Experts. https://www.ecdc.europa.eu/en/disease-vectors/facts/mosquito-factsheets/aedes-koreicus.

[11] Go Y.Y., Balasuriya U.B.R., Lee C.-K. (2014). Zoonotic encephalitides caused by arboviruses: Transmission and epidemiology of alphaviruses and flaviviruses. Clin. Exp. Vaccine Res..

[12] Tsai T.F., Popovici F., Cernescu C., Campbell G.L., Nedelcu N.I. (1998). West Nile encephalitis epidemic in southeastern Romania. Lancet.

[13] Young J.J., Haussig J.M., Aberle S.W., Pervanidou D., Riccardo F., Sekulić N., Bakonyi T., Gossner C.M. (2021). Epidemiology of human West Nile virus infections in the European Union and European Union enlargement countries, 2010 to 2018. Eurosurveillance.

[14] https://www.ecdc.europa.eu/sites/default/files/documents/Surveillance_prevention_and_control_of_WNV_and_Usutu_virus_infections_in_the_EU-EEA.pdf.

[15] Habarugira G., Suen W.W., Hobson-Peters J., Hall R.A., Bielefeldt-Ohmann H. (2020). West nile virus: An update on pathobiology, epidemiology, diagnostics, control and “One health” implications. Pathogens.

[16] Orton S., Stramer S., Dodd R. (2005). Self-reported symptoms associated with West Nile virus infection in RNA-positive blood donors. Transfusion.

[17] Huhn G.D., Dworkin M.S. (2006). Rash as a prognostic factor in West Nile Virus disease. Clin. Infect. Dis..

[18] Zou S., Foster G.A., Dodd R.Y., Petersen L.R., Stramer S.L. (2010). West Nile fever characteristics among viremic persons identified through blood donor screening. J. Infect. Dis..

[19] Lim J.K., McDermott D.H., Lisco A., Foster G.A., Krysztof D., Follmann D., Stramer S.L., Murphy P.M. (2010). CCR5 deficiency is a risk factor for early clinical manifestations of west nile virus infection but not for viral transmission. J. Infect. Dis..

[20] Sutinen J., Fell D.B., Sander B., Kulkarni M.A. (2022). Comorbid conditions as risk factors for West Nile neuroinvasive disease in Ontario, Canada: A population-based cohort study. Epidemiol. Infect..

[21] Patel H., Sander B., Nelder M.P. (2015). Long-term sequelae of West Nile virus-related illness: A systematic review. Lancet Infect. Dis..

[22] Popescu C.P., Florescu S.A., Hasbun R., Harxhi A., Evendar R., Kahraman H., Neuberger A., Codreanu D., Zaharia M.F., Tosun S. (2020). Prediction of unfavorable outcomes in West Nile virus neuroinvasive infection—Result of a multinational ID-IRI study. J. Clin. Virol..

[23] Sejvar J.J. (2014). Clinical Manifestations and outcomes of West Nile virus infection. Viruses.

[24] Kramer L.D., Li J., Shi P.-Y. (2007). West Nile virus. Lancet Neurol..

[25] Ali M., Safriel Y., Sohi J., Llave A., Weathers S. (2005). West Nile Virus infection: MR imaging findings in the nervous system. Am. J. Neuroradiol..

[26] Herring R., Desai N., Parnes M., Jarjour I. (2019). Pediatric West Nile Virus-Associated Neuroinvasive Disease: A Review of the Literature. Pediatr. Neurol..

[27] Li J., Loeb J.A., Shy M.E., Shah A.K., Tselis A.C., Kupski W.J., Lewis R.A. (2003). Asymmetric flaccid paralysis: A neuromuscular presentation of West Nile virus infection. Ann. Neurol..

[28] Garg S., Jampol L.M. (2005). Systemic and intraocular manifestations of West Nile virus infection. Surv. Ophthalmol..

[29] Frasca F., Sorrentino L., Fracella M., D’auria A., Coratti E., Maddaloni L., Bugani G., Gentile M., Pierangeli A., D’ettorre G. (2024). An Update on the Entomology, Virology, Pathogenesis, and Epidemiology Status of West Nile and Dengue Viruses in Europe (2018–2023). Trop. Med. Infect. Dis..

[30] Pavesi A., Tiecco G., Rossi L., Sforza A., Ciccarone A., Compostella F., Lovatti S., Tomasoni L.R., Castelli F., Quiros-Roldan E. (2024). Inflammatory Response Associated with West Nile Neuroinvasive Disease: A Systematic Review. Viruses.

[31] European Centre for Disease Prevention and Control, An Agency of the European Union Local Transmission of Dengue Virus in Mainland EU/EEA, 2010–Present. https://www.ecdc.europa.eu/en/all-topics-z/dengue/surveillance-and-disease-data/autochthonous-transmission-dengue-virus-eueea.

[32] (2024). WHO position paper on dengue vaccines—May 2024. Weekly Epidemiological Record.

[33] Gwee S.X.W., John A.L.S., Gray G.C., Pang J. (2021). Animals as potential reservoirs for dengue transmission: A systematic review. One Health.

[34] Nishiura H., Mizumoto K., Ejima K., Yamamoto T. (2014). On the risk of severe dengue during secondary infection: A systematic review coupled with mathematical modeling. J. Vector Borne Dis..

[35] Trivedi S., Chakravarty A. (2022). Neurological Complications of Dengue Fever. Curr. Neurol. Neurosci. Rep..

[36] Carod-Artal F.J., Wichmann O., Farrar J., Gascón J. (2013). Neurological complications of dengue virus infection. Lancet Neurol..

[37] Pandey A., Verma R., Jain A., Prakash S., Garg R.K., Malhotra H.S., Sharma P.K., Kumar N., Uniyal R., Pandey S. (2021). Correlation of serotype-specific strain in patients with dengue virus infection with neurological manifestations and its outcome. Neurol. Sci..

[38] Wu S., Zhang T., Qiang W., Yang Y. (2024). Modulation of immune responses in the central nervous system by Zika virus, West Nile virus, and dengue virus. Rev. Med. Virol..

[39] Noh M.S.F.M., Rashid A.M.A., Zaidi W.A.W., Khoo C.S., Rajadurai N., Muda A.S. (2018). Neuroimaging in dengue: CT and MRIfeatures. Neurol. Clin. Neurosci..

[40] European Centre for Disease Prevention and Control (2024). Communicable Disease Threats Report.

[41] https://www.ecdc.europa.eu/sites/default/files/documents/CHIK_AER_2022_report.pdf.

[42] https://www.ecdc.europa.eu/en/chikungunya-virus-disease/surveillance-and-updates/seasonal-surveillance.

[43] Caron M., Paupy C., Grard G., Becquart P., Mombo I., Nso B.B.B., Kassa F.K., Nkoghe D., Leroy E.M. (2012). Recent introduction and rapid dissemination of Chikungunya virus and Dengue virus serotype 2 associated with human and mosquito coinfections in Gabon, Central Africa. Clin. Infect. Dis..

[44] Parola P., de Lamballerie X., Jourdan J., Rovery C., Vaillant V., Minodier P., Brouqui P., Flahault A., Raoult D., Charrel R. (2006). Novel Chikungunya virus variant in travelers returning from Indian Ocean islands. Emerg. Infect. Dis..

[45] Gérardin P., Barau G., Michault A., Bintner M., Randrianaivo H., Choker G., Lenglet Y., Touret Y., Bouveret A., Grivard P. (2008). Multidisciplinary prospective study of mother-to-child Chikungunya virus infections on the island of La Réunion. PLoS Med..

[46] Burt F.J., Rolph M.S., E Rulli N., Mahalingam S., Heise M.T. (2012). Chikungunya: A re-emerging virus. Lancet.

[47] Consuegra-Rodríguez M.P., Hidalgo-Zambrano D.M., Vásquez-Serna H., Jimenez-Canizales C.E., Parra-Valencia E., Rodriguez-Morales A.J. (2018). Post-chikungunya chronic inflammatory rheumatism: Follow-up of cases after 1 year of infection in Tolima, Colombia. Travel Med. Infect. Dis..

[48] Brizzi K. (2017). Neurologic Manifestation of Chikungunya Virus. Curr. Infect. Dis. Rep..

[49] da Costa V.G., Saivish M.V., Sinhorini P.F., Nogueira M.L., Rahal P. (2024). A meta-analysis of Chikungunya virus in neurological disorders. Infect. Dis. Now.

[50] Puccioni-Sohler M., Soares C.N., Christo P.P., de Almeida S.M. (2023). Review of dengue, zika and chikungunya infections in nervous system in endemic areas. Arq. Neuro-Psiquiatr..

[51] Robin S., Ramful D., Le Seach F., Jaffar-Bandjee M.-C., Rigou G., Alessandri J.-L. (2008). Neurologic Manifestations of Pediatric Chikungunya Infection. J. Child Neurol..

[52] Cerny T., Schwarz M., Schwarz U., Lemant J., Gérardin P., Keller E. (2017). The Range of Neurological Complications in Chikungunya Fever. Neurocrit. Care.

[53] Silva C.N.P., Crispim J.G., Pereira M.C., Pitta M.G.d.R., Rêgo M.J.B.d.M., da Rosa M.M. (2025). The communication between chikungunya infection and the central nervous system. Microb. Pathog..

[54] Fraiman P.H.A., Freire M., Fernandes B., Palitot F., Mota N., Sequerra E., Santos G., Dourado M.E., Godeiro-Junior C.d.O., Moreira-Neto M. (2024). “Clock dial pattern”, a radiologic clue to neuro-chikungunya diagnosis: A case series | ‘Padrão em ponteiro de relógio’, uma dica radiológica para o diagnóstico de neurochikungunya: Série de casos. Arq. Neuro-Psiquiatr..

